# Antibacterial mechanism of action and in silico molecular docking studies of *Cupressus funebris* essential oil against drug resistant bacterial strains

**DOI:** 10.1016/j.heliyon.2023.e18742

**Published:** 2023-07-27

**Authors:** Caixin Yuan, Xiuqiao Hao

**Affiliations:** a Department of Supply Room, The Fourth Hospital of Hebei Medical University, No. 12 Jiankang Road, Shijiazhuang City, Hebei Province, 050011, PR China; b Department of Hematology, The Fourth Hospital of Hebei Medical University, No. 12 Jiankang Road, Shijiazhuang City, Hebei Province, 050011, PR China

**Keywords:** Essential oil, Cupressus funebris, Multiple-drug resistance (MDR), Molecular docking, DNA gyrase-B

## Abstract

The primary objective of this research work was to study the antibacterial effects of *Cupressus funebris* essential oil (EO) against various drug resistant bacterial pathogens along with studying the molecular docking interactions of the major components of the EO with the key bacterial proteins/enzymes. Gas chromatography-mass spectrometry was used to analyse the chemical composition of the *Cupressus funebris* EO. The initial antibacterial screening was performed by using disc diffusion and microdilution methods. Scanning electron microscopy was also performed in order to study effects of the EO on bacterial cell morphology. Further, molecular docking studies were performed using Autodock Vina and results were visualised by BIOVIA Discovery Studio. The chemical composition of the EO showed the presence of 15 components with citronellal, terpinene-4-ol, α-phellandrene and 1,8-cineole as the major components of the EO. Results indicated that the EO of *Cupressus funebris* exhibited dose-dependent as well as time dependent antibacterial effects. The scanning electron microscopy indicated that the *Cupressus funebris* EO led to membrane rupture and permeabilization of the bacterial cells. Molecular docking studies indicated that the major compounds of the EO (citronellal and terpinene-4ol) showed strong interactions with the active site of the bacterial DNA gyrase enzyme explaining the antibacterial mode of action of the EO. Ciprofloxacin was also used for docking which showed stronger interactions with the target protein than citronellal or terpinene-4-ol. In conclusion, the major findings of the current study were that the EO of *Cupressus funebris* causes bacterial membrane rupture and permeabilization, shows time-dependent and dose-dependent antibacterial action, along with interacting with crucial bacterial enzyme viz., DNA gyrase as indicated by molecular docking studies.

## 1 Introduction

Infectious diseases contribute to a significant share of deaths and impairments worldwide. Public health is at risk from a number of factors, including the development of novel resistant microbial phenotypes and the spread of illnesses caused by MDR organisms. It is anticipated that by 2050, 10 million people would have lost their lives owing to infections that are resistant to several treatments, with 700,000 deaths occurring annually as a result of such diseases over the globe [1,2]. Notwithstanding the uncertainties surrounding this estimate, one of the biggest problems in medicine today is antibiotic resistance. Antibiotic abuse and misuse, self-drug addiction, incomplete antibiotic courses, nosocomial infections, antibiotic use in veterinary settings, and incorrect disposal of hazardous biomedical waste are some of the leading sources of antimicrobial resistance [3].

What's more worrisome is that certain strains have become immune to almost all of the common therapies. Methicillin-resistant *Staphylococcus aureus* (MRSA) is a particularly egregious example of this phenomenon due to the fact that it is immune to not just methicillin (created to fight *S. aureus* strains resistant to penicillin) but other antibiotics as well including lincosamides, chloramphenicol, tetracycline, macrolides, and aminoglycosides. Since certain strains of MRSA remain immune to antiseptics, they may play a substantial role in the spread of infections acquired in hospitals. An antiquated antibiotic called vancomycin has made a return in the fight against MRSA infections. Vancomycin resistance is widespread in Enterococcus and spread to MRSA in 2002; nevertheless, MRSA strains with acquired resistance are still rare [4]. The spread of gram-(−) bacteria which remain immune for almost all antimicrobials is even a major health problem [5].

EOs are a diverse group of plant-based specialised metabolic compounds that aid plants in resisting environmental stressors, including microbial diseases. EOs are intricate mixtures of tiny volatile chemicals with many well-established uses like in the crop protection sectors, cosmetics, culinary, medicinal, and textile [[6], [7], [8], [9], [10], [11]]. Many investigations have been conducted, leading to a plethora of articles in the medical literature, because of their broad-spectrum antibacterial action [[12], [13], [14]]. In contrast to the narrow spectrum of action of traditional antibiotics, EOs have a broader spectrum of activity against MDR bacteria with multi-target action and a little probability of microbial resistance due to their complex chemical makeup [15].

The *Cupressus funebris* Endl. Tree grows in Northern Vietnam and Central China, which is used to produce Chinese cedarwood oil. In accordance with the widely-referenced French standard AFNOR, The existence of four characteristic components distinguish commercial Chinese cedarwood, among them are thujopsene, α-cedrene, β-cedrene, and cedrol (> 10%) [16]. Only a small number of research have revealed the chemical profile of the EO extracted from *C. funebris* wood, as noted by Lawrence [17]. Significant amounts of the sesquiterpenes including α-cedrene (20.5% and 26.4%), widdrol (1.4 and 9.5%), β-cedrene (5.0 and 9.2%), thujopsene (29.9 and 31.7%), and cedrol (9.6 and 10.4%), may be found in Chinese *C. funebris* wood EO [18,19]. Recent ^13^C NMR investigation of a Vietnamese wood oil has shown the presence of α-cedrene (39.1%), cedrol (18.2%), β-cedrene (10.5%), thujopsene (7.8%), cuparene (3.7%), β-funebrene (3.0%), and α-funebrene (2.4%) [20]. Apart from wood oil, the main components of *C. funebris* leaf oil are the monoterpenes limonene, sabinene, terpinen-4-ol, α-pinene, and β-pinene [[21], [22], [23]].

The present research was set out to identify the phytocomposition and antibacterial activities of active components of *Cupressus funebris* against drug-resistant bacteria. The active components were further investigated by molecular docking experiments for their interactions with the DNA gyrase enzyme. The main purpose of using molecular docking studies is to identify the main bioactive components of the essential oil by docking them separately onto the active site of the target protein. This will help us in understanding the molecular mechanism of action of the essential oil against that particular bacterial strain. Molecular docking will confirm whether the bioactive components of the essential oil target the same active site on enzyme as ciprofloxacin or other antibiotics hinting towards its mechanism of action.

## 2 Materials and methods

### 2.1 EO extraction

The extraction of the EO from *Cupressus funebris* was accomplished with the help of the supercritical CO_2_ fluid technique. The total amount of oil extracted is influenced by a number of different factors. The optimal conditions include an extraction duration of 100 min, a pressure of 18 MPa, and a temperature of 40 °C. The EO was maintained in dark, foil-wrapped, hermetically sealed vials at a temperature of 4 °C before being analyzed. The EO collected had a particular aroma and it was light yellow in colour.

### 2.2 Phytochemical composition analysis

The EO was analyzed using a Shimadzu GC-2010 Plus gas chromatograph and GC/MS-QP 2010 Ultra System (both from Shimadzu, Kyoto, Japan). The Rxi-5MS capillary column from Restek, Bellefonte, USA, was used with a film thickness of 0.25 μm and measured 30 × 0.25 mm. Helium was employed as the carrier gas at a flow rate of 1 mL/min, and the following temperature programme was utilised: 60 °C for 1 min, increased to 280 °C at a rate of 3 °C/min, and sustained for 5 min. The injection time for the samples was 1 min at a split ratio of 1/40 and a temperature of 250 °C. In the experiment, equipment was employed that had an electron ionisation energy spectrum mass scan range of 30–500 m/z and an electron impact ionisation energy of 70 eV.

### 2.3 Antibacterial testing

#### 2.3.1 Bacterial culture and strains

The efficiency of the EO as an antibacterial agent was evaluated using a total of six distinct types of microorganisms including *E. coli* (ATCC 12344), *Haemophilus influenzae* (ATCC 33391), *Klebsiella pneumon*iae ATCC46117, Methicillin resistant *Staphylococcus aureus* (ATCC6538), *Pseudomonas aeruginosa* (ATCC27853), and *Streptococcus faecalis* (ATCC19115). The temperature of these microorganisms was maintained at 4 °C with the help of liquid paraffin wax. Thereafter, all of the strains were grown on Mueller-Hinton agar at a temperature of 37 °C.

#### 2.3.2 Disc-diffusion method

For this study, we evaluated the EO's antibacterial properties using a modified version of the paper-disk agar diffusion method described by Zeer et al. [24]. The test microorganisms were spotted onto solid MH medium plates (0.1 mL, 105 cells per mL). The EO (25 mg/mL) was impregnated into paper discs (8 mm in diameter) and then placed on the inoculation plates. These plates were kept in a 37 °C incubator for 24 h. Vernier callipers were used to measure the diameter of the inhibitory zones (DIZ). As negative controls, DMSO solution was utilised, and as a positive control, penicillin, amoxycillin, and ciprofloxacin (2.5 g/mL) were employed.

#### 2.3.3 Microdilution

By using the agar dilution technique, the EO's MIC and MBC values were evaluated. To reach a concentration of 25 mg/mL, the EO of *Cupressus funebris* was dissolved in DMSO (2%), then added to the 20 mL sterile nutritional broth (NB) medium. The concentrations of the EO used for testing ranged from 25 to 1.2 mg/mL. Next 100 mL of bacterial suspensions (adjusted to 1 × 10^5^ CFU/mL) were added to each plate. The plates were incubated at 37 °C for 24 h. Three duplicates of each experiment were carried out.

#### 2.3.4 Growth curve analysis

The growth-curve approach was used in the course of the investigation of the EO's antibacterial effectiveness. EO was put through tests against MRSA at 1MIC, 2MIC, and 1% DMSO serving as a control. After that, the bacteria were allowed to grow while shaking at 200 rpm at a temperature of 37 °C for a total of 0–3 h, 3–6 h, 9–12 h, 15–18 h, 21–24 h, and 24 h. A UV–Vis spectrophotometer was used in order to ascertain the optical density at 600 nm (OD_600_) of the supernatants at a number of different intervals. There were three separate measurements taken for each item. The exponential growth rate of MRSA was measured with the use of the assay described above. The supernatant's optical density at 600 nm (OD_600_) was plotted along the vertical axis, while time was shown along the horizontal axis (ranging from 0 to 24 h).

#### 2.3.5 Protein leakage assay

The state of the cell was watched by monitoring the amount of protein discharged into the supernatant. The Bradford assay was used in order to determine the relative levels of protein found in the supernatants [25]. EO was added to logarithmically developing MRSA bacterial cells at concentrations of one and two folds of MIC, respectively. After that, the samples were stored in an incubator at a temperature of 37 °C for one, six, twelve and 24 h respectively. After being centrifuged at a speed of 12,000 rpm for 10 min at a temperature of 4 °C, the bacteria were sorted. To determine the rate of cytoplasmic protein release, a measurement of the optical density in the supernatant taken at 595 nm was carried out.

#### 2.3.6 Scanning electron microscopy (SEM) analysis

The use of a scanning electron microscope was required in order to see the morphological changes that occurred in the target bacteria cells. At a temperature of 37 °C and for a period of 12 h, MRSA were cultured in NB medium. In addition to a control group, cells of MRSA that were in the logarithmic development phase were given a treatment with the EO at a dose of 2 MIC. At a temperature of 37 °C, the samples were left to incubate for 3, 9, and 24 h, respectively. Centrifuged at 12,000 g for 10 min at 4 °C, bacteria were then treated with glutaraldehyde at a volume-to-volume ratio of 2.0% for 3 h (pH 7.2, 2 times). The cells were then fixed with 1% osmic acid at 4 °C for 1.5 h, and then washed with 0.1 M PBS after centrifugation (pH 7.2). After this step, the cells were dehydrated in ethanol solutions varying in concentration from 50% to 100%. Before the samples were analyzed using a SEM (Hitachi S-3000H purchased from Hitachi Ltd. in Tokyo, Japan), a gold 157 vacuum sputter coating was placed to each of them.

#### 2.3.7 Molecular docking studies

To perform molecular docking simulations of selected legends into the active binding sites of protein, the AutoDock Vina software was used. The ChemOffice tool known as “Chem Draw 16.0″ was used to demonstrate the correct 2D orientation of the chemical structures of the selected ligands, and ChemBio3D was utilised to reduce the amount of energy for each molecule. In order to conduct a docking simulation in AutoDock Vina, the molecules with the least amount of energy were selected. By searching through the protein data bank repository, the three-dimensional structure of the receptor protein was downloaded as pdb format with a PDB ID of 6F86. In order to successfully prepare the target-protein in accordance with standard Protocol [26], it was necessary to liberate the co-crystallized ligand, as well as certain water molecules and cofactors. Finally, Auto Preparation of target protein file Auto Dock 4.2 (MGL tools1.5.6) was then used to create the final file for uploading to AutoDock Vina, keeping the relevant residue with protein intact. In the graphical user interface application, the docking simulation grid box was given its initial configuration. Inside the grid, the target area of the macromolecule was positioned such that it would cover the whole of the structure. It was established that the ligand and protein could be docked in the most effective manner by making use of the docking approach that was made available by Auto Dock Vina. A maximum of nine conformations of each ligand were investigated while the docking procedure was being carried out. The Discovery studio visualizer was used to investigate ligand-receptor interactions in 3D and 2D. This tool chose the free binding energy conformations that were favourable (lower) for the target receptor. The residues that interact with one other and with H bonds are displayed as balls and sticks, while the ligands are shown in a variety of colours.

#### 2.3.8 Drug-likeness and in silico ADMET studies

Using the SwissADME online tool, computational predictions of citronellal and terpinen-4-ol ADME, drug-likeness, and pharmacokinetics features were made. Additionally, citronellal and terpinen-4-ol toxicity study was carried out using the pkCSM web server. The following physicochemical parameters and ADME features were determined for these two molecules which include molecular weight (MW), hydrogen bond acceptor (HBA), hydrogen bond donor (HBD), topological polar surface area (TPSA), octanol/water partition coefficient (LogP), and rotatable bond count (RB). Citronellal and terpinene-4-ol canonical smiles were entered into the ADMET/SAR programme, and results appeared shortly after submission of the canonical smile.

#### 2.3.9 Statistical analysis

The three duplicates for each experiment were run and the results were presented as mean ± SD. For statistical analysis, the Student's t-test was utilised, and *p* values were regarded as significant at *p* < 0.01.

## 3 Results

### 3.1 Phytochemical composition of *Cupressus funebris* EO

**Fig. 1:**
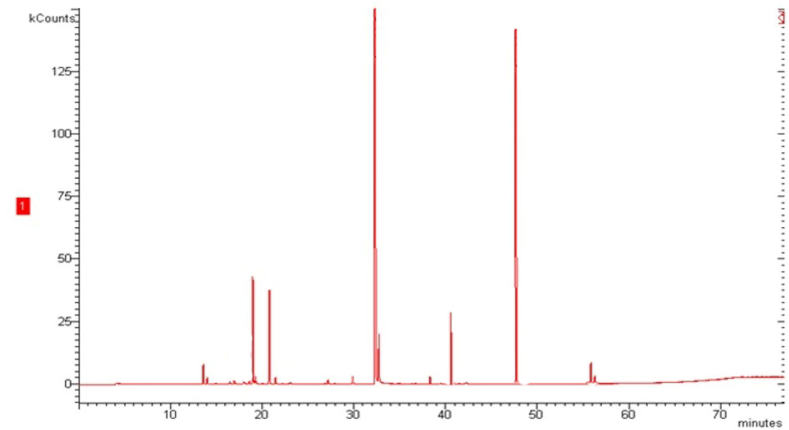
GC-MS Total Ion Chromatogram (TIC) of the leaf essential oil of *Cupressus funebris.*

The extraction of the EO was carried out with the aid of a supercritical CO_2_ fluid mechanism. The EO's molecular components were studied using a GC-MS system. The leaves of the *Cupressus funebris* tree yielded 0.5% of the oil (w/w). GC-MS was used to analyse the EO of *Cupressus funebris*, and the results revealed that the oil's composition is made up completely of fifteen separate chemical components. It was discovered that the primary constituents were Citronellal, Terpinen-4-ol, α-phellandrene, *p*-cymene, and 1, 8-cineole. One of the most common classes of chemicals identified were oxygenated monoterpenes followed by hydrocarbon monoterpenes. Fig. 1 displays the GC-MS Total Ion Chromatogram (TIC) of *Cupressus funebris* EO, and Table 1 provides a summary of the chemical components that were found in the oil.

**Table 1:** Chemical composition of essential oil from the leaves of *Cupressus funebris*.

S. No .	Compound	% Peak Area	Methods of identification
1	α-Pinene	3.5	MS, RI
2	β-Pinene	0.2	MS, RI
3	δ-3-Carene	0.5	MS, RI
4	**α-phellandrene**	**15.9**	MS, RI
5	***P*-Cymene**	**7.6**	**MS, RI, Std**
6	**1,8-Cineole**	**10.2**	**MS, RI, Std**
7	γ-Terpinene	3.9	MS, RI
8	(Z)-β-Ocimene	4.2	MS, RI
9	**Terpinen-4-ol**	**20.2**	MS, RI
10	D-Camphor	5.4	MS, RI
11	**Citronellal**	**20.5**	**MS, RI, Std**
12	Citronellol	1.3	MS, RI
13	Citronellic acid	3.3	MS, RI
14	Germacrene D	2.1	MS, RI
15	α-Bergamotene	0.4	MS, RI
			
Total		**99.2%**	

### 3.2 Antibacterial activity of the *Cupressus funebris* EO

In order to investigate the antibacterial properties of the *Cupressus funebris* EO, it was subjected to testing against a total of six distinct bacterial strains. The data for the inhibition zones of *Cupressus funebris* EO against the six selected bacterial strains are presented in Table 2. According to the results, the EO has antibacterial properties against all of the microorganisms that were put through the test, including both Gram-(+) and Gram-(−) organisms. The conventional antibiotics penicillin, amoxicillin, and ciprofloxacin were tested alongside the EO to serve as positive controls in order to determine the EO's level of effectiveness in preventing the development of bacteria. According to the results, the EO of *Cupressus funebris* has significant antibacterial capabilities that are effective against a broad variety of different microorganisms. The most effective growth inhibition against *Haemophilus influenzae* followed by MRSA was shown by EO, which had a MIC/MBC and inhibition zones of 4.2/4.8 μg/mL and 26.2 mm and 4.5/6.2 μg/mL and 10.2, respectively. *Pseudomonas aeruginosa*, on the other hand, showed the least sensitivity to the EO, with a zone of inhibition of 13.2 mm and MIC/MBC values of 8.1/7.6 μg/mL, respectively. Given that *Pseudomonas aeruginosa* is a Gram-negative bacteria, it should not come as a surprise that it has acquired the ability to withstand the majority of antibiotics. After comparing the EO's MIC and MBC values to those of standard positive controls, it was discovered that the antibacterial activity of the EO is comparable with that of the reference standards. The concentration of EO that, below which, bacterial growth could no longer be detected was used as the basis for calculating the minimum inhibitory concentration (MIC) of the oil. The minimum EO concentration resulting in the elimination of inoculum bacteria growth was taken as MBC. We were able to discover that *Haemophilus influenzae* was the bacteria that was most influenced by the EO considering the zone of inhibition as well as the MIC/MBC data.

**Table 2:** Antibacterial activity of Cupressus funebris essential oil against some common infection causing bacteria.

Bacterial strain (cat. no.)	Zone of inhibition (mm)					
	Essential oil (μg/ml)	Penicillin	Amoxycillin	Ciprofloxacin	MIC (mg/ml)	MBC (mg/ml)
*Methicillin resistant Staphylococcus aureus (MRSA) (*ATCC6538)	10.5	36.1	31.4	31.7	4.5	6.2
*E. coli (*ATCC-12344)	12.4	31.2	36.2	32.9	4.1	7.6
*Streptococcus faecalis (*ATCC19115)	16.1	34.2	26.5	24.5	5.1	9.8
*Pseudomonas aeruginosa (*ATCC27853)	13.2	25.2	22.6	23.1	8.1	7.6
*Klebsiella pneumoniae* ATCC46117	17.6	38.1	33.1	36.5	6.3	6.2
*Haemophilus influenzae* ATCC-33391	26.2	23.5	31.2	27.2	4.2	4.8

### 3.3 Results of growth curve analysis

**Fig. 2:**
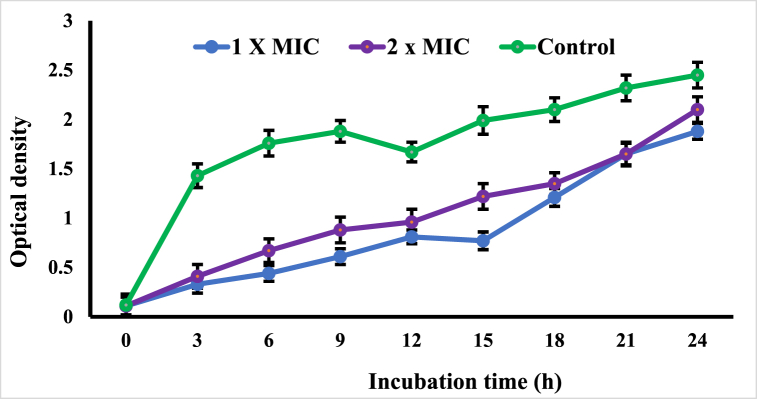
The growth curves of the *MRSA* affected by the essential oil from the leaves of *Cupressus funebris.* The results were considered significant at *p* < 0.01.

MRSA was selected as a model organism to further research in order to validate the antibacterial mechanism of *Cupressus funebris* EO based on the sensitivity of the bacteria that were utilised in the testing as well as the drug resistance shown by MRSA. Fig. 2 displays the results indicating bacteria that were not treated with EO showed a significant increase in number, in contrast to the bacteria that had been treated with EO. Nonetheless, the treatment with EO leading to a drastic drop in bacterial counts that were viable. EO reduced microbial growth throughout the course of the first 12 h of the test, the treatments (1 × MIC) did show a slight decrease in the total number of bacterial colonies. In spite of the varying pattern of bacterial counts at 1 × MIC, the number of viable cells decreased drastically during the first 3 h after culture with 2 × MIC treatment. According to these results, a wide range of concentrations of *Cupressus funebris* EO and different incubation durations were used revealing that it has a notable capacity to antibacterial potential.

### 3.4 Cupressus funebris essential oil treatment led to leakage of crucial bacterial proteins

**Fig. 3:**
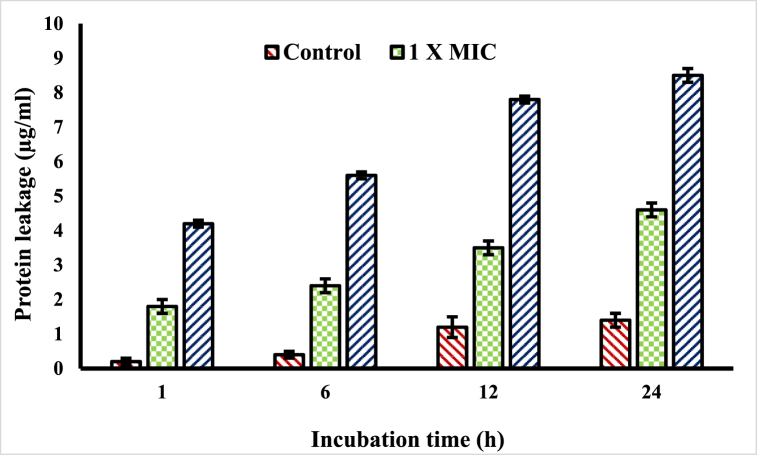
The essential oil of Cupressus funebris induces leakage of important proteins from MRSA. The results were considered significant at *p < 0.01.

In bacteria, proteins serve the key role of maintaining cellular integrity and performing key functions. In this research, the EO of *Cupressus funebris* was put to the test to see whether or not it could cause protein leakage from the MRSA bacteria. The research results shown in Fig. 3 demonstrate that the EO of *Cupressus funebris* induced the membranes of the MRSA bacterial cells to rupture, which resulted in the release of important proteins from the cells of the bacteria. In the control group, which had not been pretreated with *Cupressus funebris* EO, there was a significantly reduced amount of protein loss. After an hour of incubation, the untreated control leaked 0.2 μg/mL of protein, whereas the treated controls leaked 1.8 μg/mL (1 fold of MIC) and 4.2 μg/mL (2 fold of MIC) of protein, respectively. The treated controls also leaked more protein than the untreated control both at 1 × MIC and 2 × MIC). Protein loss was shown to be related to both the treatment time length and the quantity of EO that was used. According to the results, the EO of *Cupressus funebris* succeeded in raising the sum of protein discharge from the bacterial cells because it could enter cells via damaged membranes.

### 3.5 Scanning electron microscopic examination of the treated bacteria

**Fig. 4:**
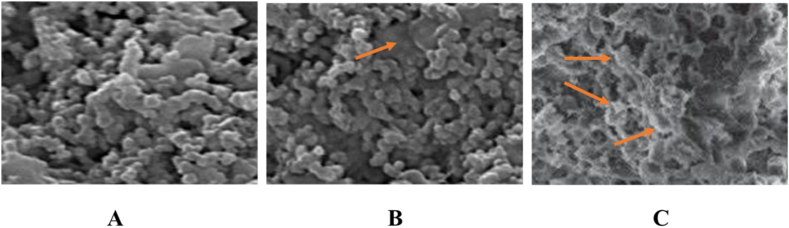
Scanning electron microscopy (SEM) images of MRSA (methicillin-resistant *Staphylococcus aureus*); A indicates untreated cells showing normal bacterial morphology, while as B indicates MIC dose of the essential oil. C indicates 2 x MIC dose of the essential oil. The arrows indicate the deformed cellular morphology and ruptured bacterial cells.

Before and after treatment with the EO, we examined the ultrastructural features of MRSA using a scanning electron microscope. When contrasted with the bacteria that were left untreated as a control, the cells of the bacteria that had been treated with EO indicated a considerable amount of cell damage (Fig. 4 (A-C)). The bacterial cells shrank, changed form, and their cell walls collapsed as a result of the EO treatment. It seems that the damage done to the cells was made worse when the dose of the EO was increased (1 X MIC and 2 X MIC). Untreated controls, on the other hand, were of a consistent size and form, taking on the appearance of circular balls that were regular and unaltered in their morphology. This further indicated that the cell wall and membrane were significantly damaged, which was already proven by the results from the study of the growth curve and the testing of the membrane integrity.

### 3.6 In silico molecular docking analysis

**Fig. 5:**
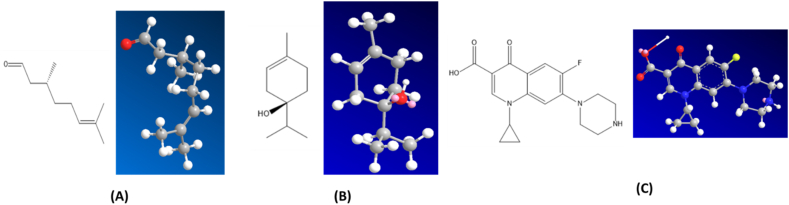
2D and 3D Structures of (A) Citronellal; PubChem CID_7794, (B) Terpinen-4-ol; PubChem CID_2724161, and (C)Ciprofloxacin; PubChem CID_2764 generated by using ChemBio3D Ultra software.

**Fig. 6:**
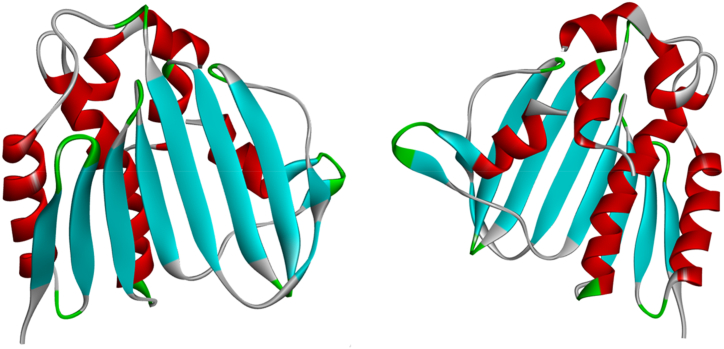
Crystal structure of DNA gyrase B (PDB: 6F86). 3D graphics was generated using Discovery Studio Visualizer 2021.

**Fig. 7:**
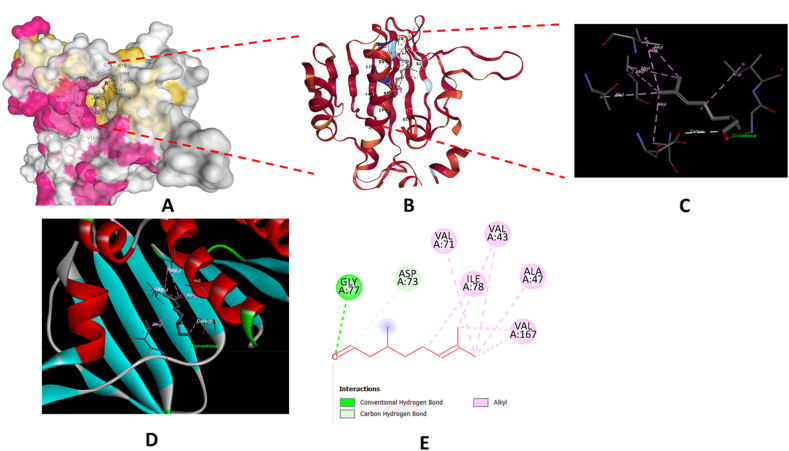
In silico analysis of the binding pattern of Citronellal with DNA-gyrase B protein target; (A) represents cartoon surface representation; (B) (C) and (D) represent Citronellal bound to the active catalytic centre of the protein target; (E) represents the 2D interaction of Citronellal with the important amino acid residues of protein target.

**Fig. 8:**
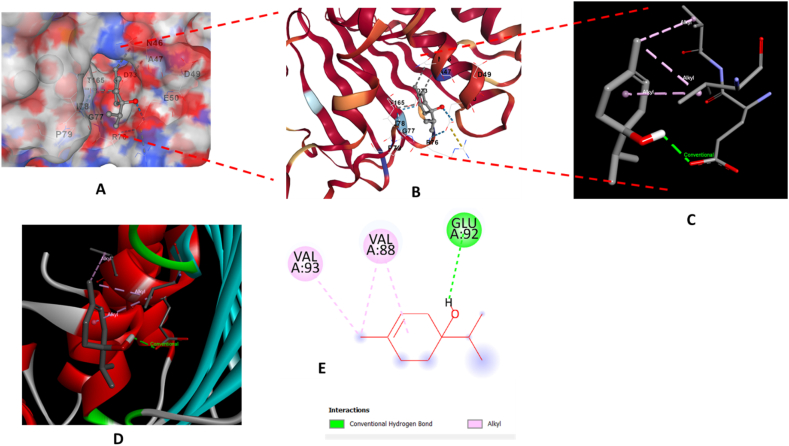
In silico analysis of the binding pattern of Terpinen-4-ol with DNA-gyrase B protein target; (A) represents cartoon surface representation; (B) (C) and (D) represent Terpinen-4-ol bound to the active catalytic centre of the protein target; (E) represents the 2D interaction of Terpinen-4-ol with the important amino acid residues of protein target.

The antibacterial activity of the major constituents of the essential oil (citronellal and terpinene-4-ol) was also investigated using molecular docking simulation studies and compared with the standard antibiotic that is ciprofloxacin. Using the AutoDock tool, citronellal, terpinene-4-ol and ciprofloxacin compounds were docked onto the active sites of DNA gyrase-B (PDB ID: 6F86) target protein in order to evaluate the antibacterial mode of action of these major compounds and compare its efficacy with ciprofloxacin. 2D and 3D models of citronellal, terpinene-4-ol and ciprofloxacin were retrieved from the PubChem database (Fig. 5 (A-C)). This docking investigation was conducted using the 3D crystal structure of DNA gyrase-B (PDB ID: 6F86) generated by Discovery Studio Visualizer 2021. The crystal structure of the target protein is shown in Fig. 6. The supposed binding sites of the target protein were evaluated indicating that the tested compounds were bound within the target protein's active binding site. The active site of the target protein was well occupied by the tested compounds especially citronellal. Fig. 7 A-E depicts the docking results of the citronellal and DNA gyrase-B receptor protein which represents cartoon surface representation, 2D and 3D graphical representation of amino acid residues involved in hydrophobic interactions and different types of bonding interactions (like hydrogen bonds, pi-alkyl, alkyl-alkyl, pi-pi, pi-cation etc) are shown by different color dotted lines. Binding interaction of citronellal with the target protein showed 1 conventional hydrogen bond (shown by green dotted lines) with GLY A:77. The other amino acid residues which were involved in carbon-hydrogen interactions and alkyl interactions with citronellal include ASP A:73, VAL A:71, VAL A:43, ILE A:78, ALA A:47, VAL A:167. It was observed that citronellal binds with the active site of the target protein with a binding energy score of −5.7 kcal/mol. Similarly, Fig. 8 A-E represents the cartoon surface representation, 2D and 3D graphical representation of the various interactions of the other major constituent of the essential oil which is terpinene-4-ol with the target protein of DNA gyrase-B enzyme. Binding interaction of terpinene-4-ol with the target protein showed one hydrogen bond with GLU A:92 amino acid residue while it showed two alkyl interactions with VAL A:88 and VAL A:93 amino acid residues. It was calculated that terpinene-4-ol binds with the active site of the target protein with a binding energy score of −5.0 kcal/mol which was very close to that of citronellal. The bonding score values for citronellal and terpinene-4-ol indicate that these molecules have a good binding efficiency with the active site of the DNA-gyrase-B target protein indicating their potential to inhibit the said enzyme and may be the active components of the essential oil of *Cupressus funebris*.

**Fig. 9:**
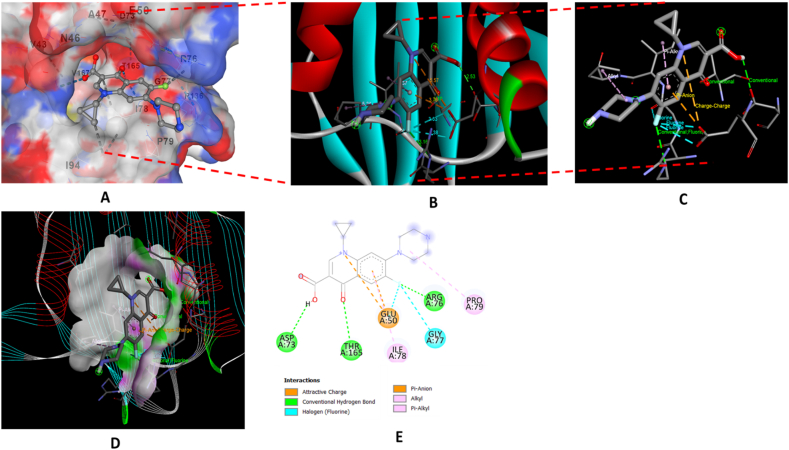
In silico analysis of the binding pattern of ciprofloxacin with DNA-gyrase B protein target; (A) represents cartoon surface representation; (B) (C) and (D) represent ciprofloxacin bound to the active catalytic centre of the protein target; (E) represents the 2D interaction of ciprofloxacin with the important amino acid residues of protein target.

**Fig. 10:**
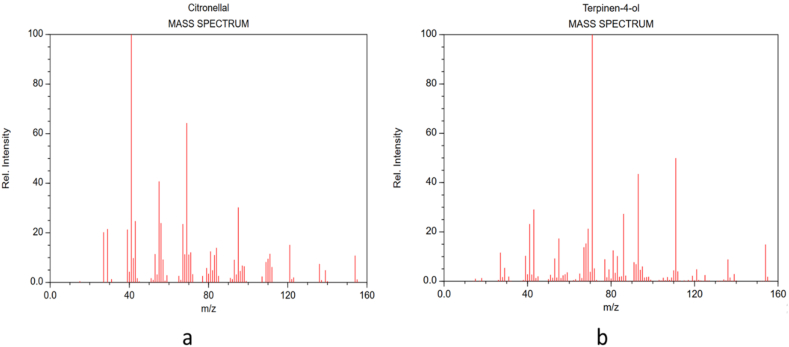
GC-MS generated mass spectra of citronellal (a) and terpinen-4-ol (b).

In order to compare the binding efficacy of citronellal and terpinene-4-ol with the target protein, we studied the binding interactions of the standard antibiotic-ciprofloxacin, with the DNA gyrase-B enzyme. Fig. 9 A-E represents the cartoon surface representation, 2D and 3D graphical representation of the various interactions of ciprofloxacin with the target protein of DNA gyrase-B enzyme. Molecular docking results indicated that ciprofloxacin showed three conventional hydrogen bonds with the ASP A:73, THR A:165 and ARG A:76 amino acid residues of the target protein. Ciprofloxacin also showed pi-anion interaction with GLU A:50 amino acid residue, halogen interaction with GLY A:77 and alkyl/pi-alkyl interactions with PRO A:79 and ILE A:78 amino acid residues. It was calculated that ciprofloxacin binds with the active site of the target protein with a binding energy score of −7.0 kcal/mol which is higher than the values of citronellal and terpinene-4-ol indicating that ciprofloxacin is a much better inhibitor of this enzyme than the active components of the essential oil. Fig. 10 (a,b) shows the mass spectra of citronellal and terpinene-4-ol which are the major components of the essential oil.

Molecular docking study is a computational and theoretical approach by means of which we can understand molecular mechanism of action of various biological and biochemical processes. This approach provides significant insights into the target protein-ligand interactions, and can express their significance in terms of binding energy values. The higher the binding energy value, the more efficient is the ligand-protein interaction. Molecular docking is highly applicable in structure-based drug designing leading to the identification of lead molecules in drug discovery. A ligand (drug molecule) can make a number of non-covalent interactions with the active sites of the target protein which include conventional hydrogen bond, alkyl-alkyl, pi-pi, pi-alkyl interactions.

Pharmacokinetic and in silico ADME parameters of citronellal and terpinene-4-ol for drug-likeness assessment:

In order to gain an understanding of the drug-likeness and pharmacokinetic aspects of citronellal and terpinene-4-ol, we used the SwissADME webserver to investigate the probable pharmacokinetic properties of these molecules. The results of the ADME analysis from the SwissADME webserver are shown in Table 3, Table 4. These metrics provide significant indications about drug absorption of a compound through cell membrane as well as provide key information about possibility of H-bond formation with target protein. The gastrointestinal (GI) absorption values of citronellal and terpinene-4-ol were high and the skin permeation values were found to be −4.52 and −4.93 cm/s respectively. The molecular weight of both citronellal and terpinene-4-ol is 154.25 g/mol which according to the drug-likeness prediction rule should be between 150 and 500 g/mol. The total polar surface area (TPSA) should be between 20 and 130 Å^2^ and in case of citronellal and terpinene-4-ol it was found to be 17.07 and 20.23 Å^2^ respectively. The average of all predicted Log po/w (Consensus Log Po/w) for citronellal and terpinene-4-ol was found to be 2.94 and 2.60 which should not be higher than 6 for drug-likeness tendency. The bioavailability score for both citronellal and terpinene-4-ol was found to be 0.55 which should not be less than 0.25 for a good drug.

**Table 3:** ADME prediction of various parameters of citronellal and terpinene-4-ol using SwissADME webserver. GI = Gastrointestinal, BBB = blood-brain barrier, Pgp = P glycoprotein, CYP = cytochrome, log Kp = skin permeability.

Compound	GI absorption	BBB permeant	Pgp substrate	CYP1A2 inhibitor	CYP2C19 inhibitor	CYP2C9 inhibitor	CYP2D6 inhibitor	CYP3A4 inhibitor	Log Kp (cm/s)
**Citronellal**	High	Yes	No	No	No	No	No	No	−4.52
**Terpinen-4-ol**	High	Yes	No	No	No	No	No	No	−4.93

**Table 4:** Drug-likeness prediction of citronellal and terpinene-4-ol using the SwissADME server. MW=Molecular weight, TPSA = total polar surface area.

Compound	MW (g/mol)	Rotatable bonds	H-bond acceptors	H-bond donors	TPSA (Å^2^)	Consensus Log P	Lipinski violations	Ghose violations	Egan violations	Veber violations	Muegge violations	Bioavailability score
**Citronellal**	154.25	5	1	0	17.07	2.94	0	1	0	0	2	0.55
Terpinen-4-ol	154.25	1	1	1	20.23	2.60	0	1	0	0	2	0.55

## 4 Discussion

The majority of serious infectious diseases are caused by bacterial infections, the most common of which being *MRSA, Streptococcus pyogenes, Bacillus anthracis, Haemophilus influenzae,* and *Streptococcus pneumoniae*, amongst others. There is evidence that besides bacteria viruses such as the coronavirus and the parainfluenza virus also contribute to serious infectious diseases [27,28]. Antibiotics have lost some of their potency as a result of the development of antibiotic resistance in several diverse sorts of bacteria. Ogston made the discovery of *S. aureus* in the 1880s when the bacteria were found in the pus of a leg abscess. This species was rapidly recognised due to their high adaptability to humans and medical environments [29]. *S. aureus* has been linked to a variety of illnesses, including endocarditis, bacteremia, osteomyelitis, skin infections, and infections of the soft tissue. *S. aureus* has emerged as a significant infectious agent that is widely dispersed across hospital settings since the beginning of modern medicine. In the 1940s, the β-lactamase gene, also known as blaZ, was discovered to be the cause of penicillin resistance for the first time. MRSA was identified after just one year of clinical usage, more than 60 years after the development of the first semi-synthetic anti-staphylococcal penicillins [30,31]. As a result, there is an urgent need for innovative antimicrobial medications that are both effective against these infections and do not quickly build resistance to them. These treatments should also be available at prices that are affordable. Since each EO has its own distinct chemical makeup, it is possible to use it in several aromatherapy and healthcare contexts. EOs have been proven to exhibit antibacterial action in a number of investigations [32], and this activity has been shown against both Gram-positive and Gram-negative bacteria. EOs and their consisting components have been used for a very long time to treat a number of conditions that affect the bacterial infections, including the common cold. Inhalation therapy with EOs has been shown to be beneficial in treating acute and chronic bronchitis, sinusitis, and even tracheal inflammation [33–26].

In this study, we investigated the antibacterial properties of *Cupressus funebris* EO and demonstrated its mode of action by utilising a number of state-of-the-art assays. These assays included protein leakage tests, growth curve assays, and scanning electron microscopy. In the mechanistic testing, the most susceptible strain of MRSA was used. The EO has strong antibacterial properties that are effective against a wide variety of bacterial strains. The development of *Haemophilus influenzae* and MRSA was inhibited by EO, which had MIC/MBC and inhibition zones of 4.2/4.8 mg/mL and 26.2 mm and 4.5/6.2 mg/mL and 10.2, respectively. *Pseudomonas aeruginosa* showed the least sensitivity to the EO based on its zone of inhibition, which was 13.2 mm, as well as its MIC and MBC values, which were 8.1 and 7.6 mg/mL, respectively. These data indicate that this bacteria was the least susceptible to the EO. Since *Pseudomonas aeruginosa* is a Gram-negative bacteria, it should not come as a surprise that it has acquired the ability to withstand treatment with the majority of antibiotics [33]. It was established that the antibacterial activity of the EO is equivalent to that of the reference strains by comparing the MIC and MBC values of the EO to those of standard positive controls. The growth curves that were used in the experiment revealed that the untreated bacteria multiplied far more quickly than the bacteria that had been exposed to the EO. The treatment with EO, on the other hand, resulted in a significant reduction in the number of live cells produced by the bacteria. Our results are in line with those of earlier research, which discovered powerful antibacterial activity in the EOs of other *Cupressus* species [34]. When EO from *Cupressus funebris* was added to the bacterial culture, the cellular components (proteins) of the bacterial cell were also released into the media. The amount of cell constituents that were released into the medium increased when greater doses of the EO were used. As a result of the treatment with EO, the cell walls broke down, the cell shapes were deformed, the cell sizes were decreased, and protein leaked out of the cells, as discovered by scanning electron microscopy. There seems to be a correlation between the dose of EO used in treatment and the level of damage caused to the bacteria. Previous studies have also reported the damage caused by the EOs to the bacterial cell wall [35].

In order to account for the molecular mechanism of action of the tested essential oil, we used in-silico molecular docking studies using two major components of the Cupressus funebris essential oil viz., citronellal and terpinene-4-ol and the target protein was chosen to be DNA-gyrase B enzyme which is the target protein for many of the clinically used antibiotics. Nalidixic acid and ciprofloxacin are well known inhibitors of DNA-gyrase B enzyme, after binding these antibiotics trap it in a transient step of the catalytic cycle resulting in cell death. On comparing the molecular docking interactions of the two major components of the essential oil viz., citronellal and terpinene-4ol with the docking interactions made by ciprofloxacin, it can be seen that ciprofloxacin binds to the active site of the enzyme much more efficiently with a much higher binding score of −7.0 kcal/mol forming three conventional hydrogen bonds with the target protein. Citronellal and terpinene-4-ol bind with the target protein with lower binding scores of −5.7 and −5.0 kcal/mol respectively each making just one hydrogen bond with the target protein. These results indicate that both citronellal and terpinene-4-ol have the tendency to bind to the active site of the protein and therefore inhibit it leading to cell death and the resulting antibacterial action of the essential oil.

The experimental results and the theoretical results (molecular docking) compliment each other well in our study as the docking studies confirm that the two main active compounds of the essential oil viz., citronellal and terpinene-4-ol have the ability to potentially inhibit the DNA-gyrase B enzyme. It is interesting to note here that this bacterial enzyme is also inhibited by clinically used antibiotics like nalidixic acid and ciprofloxacin. We docked ciprofloxacin onto the active site of the enzyme using AutoDock software and the binding energy value was found to be −7.0 kcal/mol which is quite high indicating its potency. Despite of the promising results of the current study, there are some limitations associated with the medicinal value of essential oils which include complex chemical compositions of the essential oils in which some chemical constituents can be toxic as well. Another limitation working with the essential oils is their volatile nature and they easily evaporate under experimental conditions. These limitations can be overcome by isolating single molecular species from these essential oils using column chromatography and fractional distillation. The other limitation can be overcome by incorporating these essential oils into polymeric matrices or making their nano-emulsions.

## 5 Conclusion

This study examines *Cupressus funebris* EO's chemical composition and antibacterial activity against various bacterial pathogens. *Haemophilus influenza* and *MRSA* were the key bacterial strains effected by the EO. Further investigating the mechanism of action, EO disrupted cell membranes of MRSA, releasing cellular contents including proteins. *Cupressus funebris* EO also induced significant cell morphological changes including bacterial cell wall damage in MRSA bacterial strains. In-silico molecular docking analysis indicated that the two major components of the essential oil viz., citronellal and terpinene-4ol showed strong binding with the DNA-gyrase B target protein indicating their ability to inhibit this bacterial protein leading to antibacterial action of the essential oil. The molecular docking results of these two major components were also compared with that of the standard antibiotic viz., ciprofloxacin revealing that the inhibition caused by the two active components of the essential oil (citronellal and terpinene-4ol) was comparable to the ciprofloxacin, as their binding score values were comparable. In conclusion, such studies could pave way for the design and development of potent inhibitors targeting bacterial enzymes in an effective manner. Our study can act as a pioneering study which identifies bioactive molecules from natural sources targeting a particular bacterial enzyme using in silico molecular docking analysis.

## Funding Support

Funding +Number Key Projects in 10.13039/501100015285Medical Science Research in Hebei Province (No.20190736).

## Data availability statement

Data will be made available on request.

## Author contribution statement

Caixin Yuan: Performed the experiments; Analyzed and interpreted the data; Wrote the paper, Conceived and designed the experiments. Xiuqiao Hao: Conceived and designed the experiments; Analyzed and interpreted the data; analysis tools or data; Wrote the paper.

## Declaration of competing interest

The authors declare that they have no known competing financial interests or personal relationships that could have appeared to influence the work reported in this paper.
